# A unifying force for the realization of medical AI

**DOI:** 10.1038/s41746-022-00721-7

**Published:** 2022-11-15

**Authors:** Jochen K. Lennerz, Ursula Green, Drew F. K. Williamson, Faisal Mahmood

**Affiliations:** 1 Massachusetts General Hospital/Harvard Medical School, Boston, MA USA; 2 Brigham and Women’s Hospital/Harvard Medical School, Boston, MA USA; 3 Cancer Program, Broad Institute of MIT and Harvard, Cambridge, MA USA

**Keywords:** Communication and replication, Policy

## Abstract

Artificial Intelligence (AI) in medicine has grown rapidly, yet few algorithms have been deployed. It is not the problem with the AI itself but with the way functions and results are communicated. Regulatory science provides the appropriate language and solutions to this problem for three reasons: First, there is value in the intentionally interdisciplinary regulatory language. Second, regulatory concepts are important for AI researchers because these concepts enable tackling of risk and safety concerns as well as understanding of recently proposed regulations in the US and Europe. Third, regulatory science is a scientific discipline that evaluates and challenges current regulation—aiming for evidence-based improvements. Knowledge of the regulatory language, concepts, and science should be regarded a core competency for communicating medical innovation. Regulatory grade communication will be the key to bringing medical AI from hype to standard of care. Foregoing the possible benefits of regulatory science as a unifying force for the realization of medical AI is a missed opportunity.

The past few years has seen a rapid growth of AI in medicine, however, few algorithms have been deployed in clinical practice^1^. We view this disconnect between hype and reality as stemming from two main barriers: first, the lack of a common language between AI and medicine, and second, the rapid progress in AI outpacing the comparatively slow adaptation of regulation, forcing regulatory bodies to apply measures that do not always consider the paradigm-shifting capabilities of contemporary AI. We propose regulatory science with its terms and concepts as a solution for both problems because it represents a high-level language that can serve as a unifying force for the realization of medical AI (Fig. 1).

**Fig. 1:**
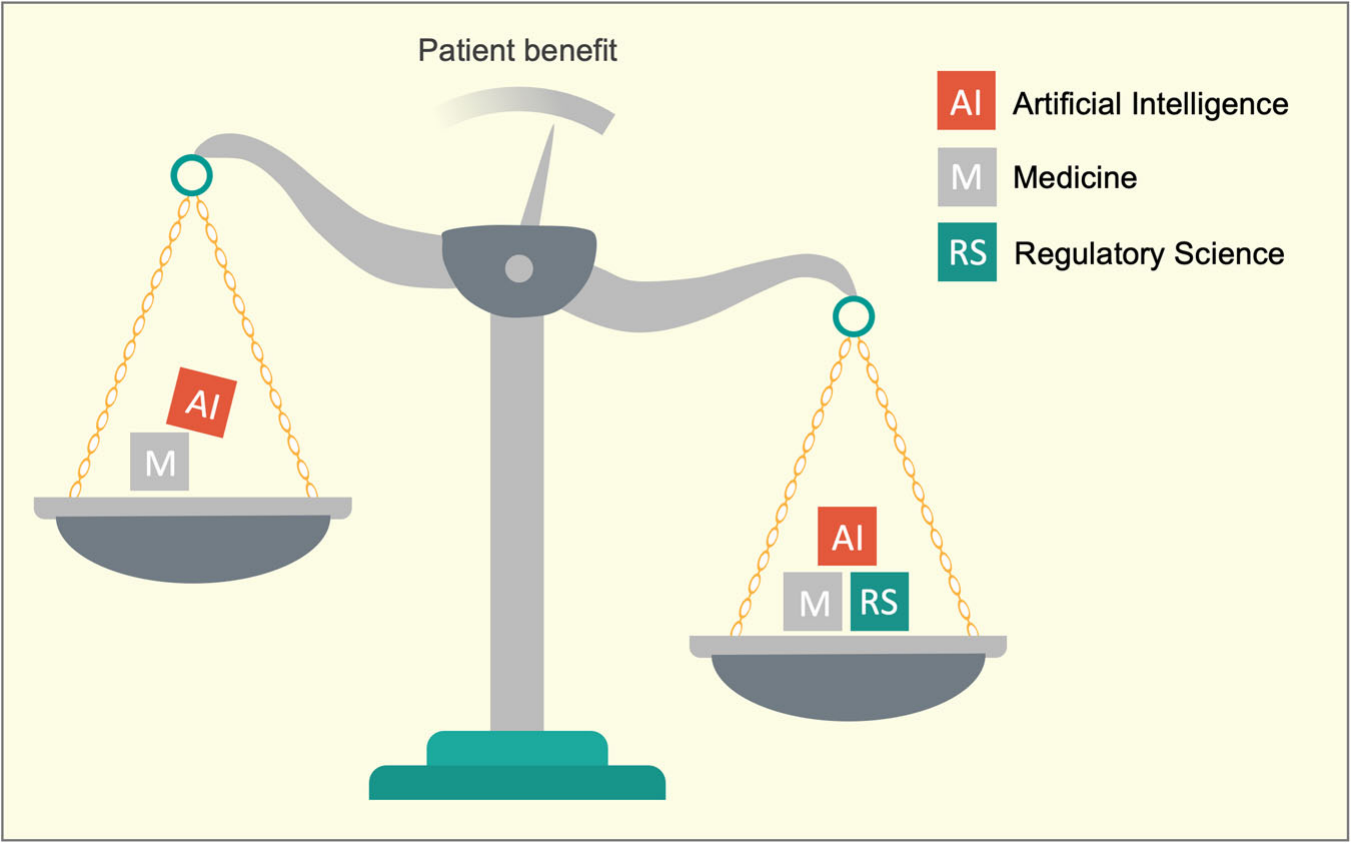
Regulatory science and AI in medicine. The application of AI in medicine aims to benefit patients. The disciplines of artificial intelligence (AI; a branch of computer science) and medicine are coexisting without a shared interdisciplinary language that enables expedient risk and benefit assessments. Regulatory science is characterized by specific and intentionally interdisciplinary language that considers multiple vantage points. Regulatory science is one proven approach to use scientific data to evaluate and challenge current regulatory paradigms and inform future regulation.

Regulatory science is the scientific discipline that *evaluates* and *challenges* current regulation, benefit vs. risk assessments, and submission/approval strategies^2^. It is the application of the scientific method to enable evidence-based improvements of regulation, and just as new scientific evidence can be powerful enough to change the paradigm of a field of study, so too can it change regulatory paradigms.

Fundamentally, regulatory science is about creating a dialogue for launching new ideas and determining how best to allow those ideas to interact with society-not only from within regulatory authorities but also through collaborations between academics, clinicians, industry, payors, policy experts, and patients. Like any scientific discipline, regulatory science comes with a specific language, but given its core translational nature, its language is intentionally interdisciplinary to enable deep collaborations. The terms and concepts traverse specific use cases and provide a contextual vocabulary that enables clear communication beyond use case of medical subspecialty (Supplementary Table 1). In other words, regulatory language is unifying.

For example, one challenge we have personally encountered (and have witnessed frequently among others) is clearly communicating the specific task of medical AI in a way that is mutually intelligible for medical and AI experts. Medical education opens one’s eyes to the enormously complex systems that have evolved for treating patients through our incomplete understanding of biology. The inherent subjectivity and guesswork in medicine can be appalling to AI experts more used to dealing with systems that are, at least in theory, rationally designed and better understood. Given the interconnectedness and subjectivity inherent in essentially all interactions a patient has with the healthcare system, defining the boundaries of a problem where AI could provide a solution becomes an issue in and of itself. For example, subtle changes in diagnosis can lead to huge changes in management. These subtleties are accounted for in the evolving and continuously updated definitions that make up the language of regulatory science. Terminology from regulatory science such as *intended use* (“what”), *indication of use* (“who and why”), or *instructions for use* (“how”); can help both sides communicate precisely about the scope of the problem at hand and how to center the patient in this discussion (Fig. 2).

**Fig. 2:**
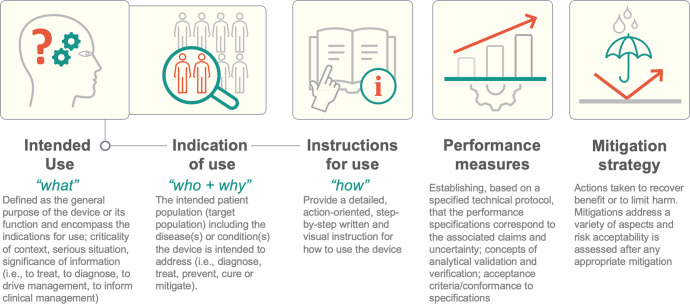
Selected regulatory science concepts. The infographic depicts 5 regulatory concepts alongside a brief explanation. Detailing these aspects provides a reasonable starting point to describe the function of a medical AI algorithm and the value of regulatory concepts for streamlining interdisciplinary communication.

Centering benefit to the patient is the goal of effective regulation, but the prevailing regulatory paradigms have not been optimized for AI in medicine. By and large, they have been adapted through continuous iteration to best review and approve drugs, medical devices, or software (as a medical device) that is fundamentally different from AI—especially when algorithms continuously evolve. A burgeoning body of research has shown that AI algorithms can fail in non-trivial ways, from poor generalization due to dataset shift, to overfitting to confounders, to unexpected failure modes^3^.

These challenges must be addressed before AI can be used safely in clinical practice. Thankfully, similar barriers have been overcome in other domains of medicine and their solutions codified into regulation. For example, there is a growing recognition that ongoing performance assessment of a deployed AI model is key to combating dataset shift, a concept that follows the principles of continued monitoring of post-market surveillance required by the FDA. There are numerous regulatory resources (Supplementary Table 1)^4^ to address software, medical AI, and change modifications^5–8^. Much additional work is needed though, with the prevailing FDA regulations (Supplementary Table 1) or ISO governance approaches (Supplementary Table 2) dispersed across over 25 guidance^2^ or standard documents, respectively.

One key question is whether applying regulatory paradigms can supplement the more traditional strength/weaknesses approach pursued in research. We have reconstructed examples where the addition or regulatory principles resulted in documented improvements (Supplementary Table 3). Briefly, the *IBM Watson Content Analytics* had a poorly described *intended use*; however, subsequent publications clearly communicate value propositions in regulatory terms (Supplementary Table 3). Google’s AI-screening for diabetic retinopathy is an example where the lack of *instructions for use* was responsible for key performance issues (e.g., operating the device in a dark room). Notably, the lack of regulatory aspects was in direct contradiction to simultaneously published regulatory comments from the FDA and (notably) google itself—emphasizing the importance of regulatory consistencies (Supplementary Table 3). In other words, we can reconstruct that two of the most drastic AI fiascoes entailed inconsistencies in communication that resulted in miscommunication between AI and healthcare experts. Other examples include documented improvements in objectivity and reproducibility when tailoring performance measures to the specific *target population*. Notably, adoption of the algorithm based on the *target population*-matched (as a *mitigation strategy*) enabled overcoming a biomarker challenge in ovarian cancer screening previously flagged as a public health concern (Supplementary Table 3). These examples illustrate that regulatory concepts are consequential and hold clinical value beyond a vantage point in a research publication.

The unique strengths and weaknesses of AI require new regulation to be developed and old regulation to be altered. For example, US-based regulatory guidances and the European Artificial Intelligence Act^9^ already account for regulatory compliant reporting of change protocols (Supplementary Table 1), a change that accounts for potential problems identified during and after deployment of continuously learning AI models. These guidance and legislative axioms argue strongly for a role of regulatory terminology as one of the key factors impacting the integration of AI approaches in medicine. Learning the language of regulatory science also confronts us with the fact that regulation, rather than being handed down from on high, is a human endeavor; that regulations are made by people who are reviewing the data and input that AI and medical experts generate, and that regulation can (and should) be challenged and updated. In the US, the FDA established several strategies to address regulatory challenges by obtaining external, interdisciplinary input (Supplementary Table 4). These programs offer concrete and practical approaches to incorporate inputs from the technical communities. For example, the FDA engages with outside experts via collaborative communities, a network of experts, and specific medical device development tool programs, to keep up with changes in the fields under its purview. Concretely, these initiatives have already influenced recent legislative proposals that now clearly spell out the need for “recommendations and other advice” from domain-experts to facilitate meaningful regulatory guidance^10^. Learning the language of regulatory science can help those who know the most about medical AI to effectively influence the nascent regulatory landscape.

We view regulatory science as a fundamental building block of healthcare that now also focusses on using AI to improve patients’ lives. Regulatory science, its language and concepts have the potential to facilitate communication and collaboration between the fields of AI and medicine, as well as between the broader medical AI community and regulatory bodies. Knowledge of the regulatory language, concepts, and science should be regarded a core competency for communicating medical innovation. Regulatory grade communication will be the key to bringing medical AI from hype to standard of care.

## Supplementary information

Supplementary Tables
