# Supplementary material for: A unifying force for the realization of medical AI
Source: NPJ Digit Med. 2022 Nov 15;5:172. doi: 10.1038/s41746-022-00721-7 (PMC9666657; doi:10.1038/s41746-022-00721-7)
Supplement: Supplementary file 1 — Supplementary Tables [file 41746_2022_721_MOESM1_ESM.pdf]

NPJDIGITALMED
Supplementary Information

Lennerz et al.,

A Unifying Force for the Realization of Medical AI

Supplementary Tables

**Page 2**	**Supplementary Table 1.** Selected Regulatory Science Concepts Relevant to Artificial Intelligence and Software as a Medical Device
**Page 3**	**Supplementary Table 2.** Selected ISO Governance Approaches for Regulating Artificial Intelligence
**Page 4**	**Supplementary Table 3.** Examples of AI-tools where the Addition of Regulatory Language and/or Concepts Resulted in Documented Improvements
**Page 5**	**Supplementary Table 4.** Established Interdisciplinary Strategies to Address Regulatory Challenges

Supplementary Table 1. Selected Regulatory Science Concepts Relevant to Artificial Intelligence and Software as a Medical Device

Concept	Abbreviated Explanation	Reference
General Principles	General Principles of Software Validation	https://www.fda.gov/regulatory-information/search-fda-guidance-documents/general-principles-software-validation<br>https://www.regulations.gov/docket/FDA-1997-D-0029
Substantial equivalence	The 510(k) Program: Evaluating Substantial Equivalence in Premarket Notifications [510(k)]	Link to guidance
Benefit Risk Assessments	Factors to Consider Regarding Benefit-Risk in Medical Device Product Availability, Compliance, and Enforcement Decisions	https://www.fda.gov/regulatory-information/search-fda-guidance-documents/factors-consider-regarding-benefit-risk-medical-device-product-availability-compliance-and
Instructions for use (IFU)	IFU should contain detailed, action-oriented, step-by-step written and visual instructions provide	https://www.fda.gov/regulatory-information/search-fda-guidance-documents/instructions-use-patient-labeling-human-prescription-drug-and-biological-products-content-and-format
CDRH Labeling Regulatory Requirements for Medical Devices	The U.S. Food and Drug Administration (FDA) develops and administers regulations under authority granted by laws passed by Congress that apply to food, drugs, cosmetics, biologics, radiation-emitting electronic products, and medical devices. Labeling regulations pertaining to medical devices are found in the following Parts of Title 21 of the Code of Federal Regulations (CFR).	https://www.fda.gov/medical-devices/overview-device-regulation/device-labeling
	Older but highly informative guidance document	https://www.fda.gov/media/74034/download
	Device Labeling	https://www.fda.gov/regulatory-information/search-fda-guidance-documents/guidance-medical-device-patient-labeling
Medical Device Labeling	General Device Labeling	21 CFR Part 801
	Use of Symbols	21 CFR Part 801.15
	In Vitro Diagnostic Products	21 CFR Part 809
	Investigational Device Exemptions	21 CFR Part 812
	Unique Device Identification	21CFR Part 830
	Good Manufacturing Practices	21 CFR Part 820
	General Electronic Products	21 CFR Part 1010
Performance Assessment (example)	Technical Performance Assessment of Quantitative Imaging in Radiological Device Premarket Submissions	https://www.fda.gov/regulatory-information/search-fda-guidance-documents/technical-performance-assessment-quantitative-imaging-radiological-device-premarket-submissions
Definition of label	Section 201(k)	https://www.fda.gov/medical-devices/overview-device-regulation/device-labeling
Medical Devices Technical Corrections Act (MDTCA)	Corrections and explanations	www.fda.gov/cdrh/mdufma/hrpt108-433.pdf
Off-the Shelf Software	OTS Software in a medical device allows the manufacturer to concentrate on the application software needed to run device-specific functions.<br>OTS Software intended for general-purpose computing may not be appropriate for a given specific use in a medical device. The medical device manufacturer using OTS Software generally gives up software life cycle control, but still bears the responsibility for the continued safe and effective performance of the medical device.	https://www.fda.gov/regulatory-information/search-fda-guidance-documents/shelf-software-use-medical-devices<br>https://www.regulations.gov/docket/FDA-2019-D-3598
Software Consensus Standards	Database provides the most up-to-date list of voluntary consensus standards to which FDA will accept a Declaration of Conformity	https://www.accessdata.fda.gov/scripts/cdrh/cfdocs/cfstandards/search.cfm<br>e.g., ISO 14971; AAMI SW68; DICOM<br>https://www.dicomstandard.org/
Quality System Regulation	requirements for the establishment and maintenance of a quality management system	21 CFRpart820; ISO 13485:2016;<br>https://www.fda.gov/medical-devices/postmarket-requirements-devices/quality-system-qs-regulationmedical-device-good-manufacturing-practices
Verification	Defined as means confirmation by examination and provision of objective evidence that specified requirements have been fulfilled. Walk-throughs, Various static and dynamic analyses, Code and document inspections, Module and level testing, Integration testing. Documentation	21CFR820.3(aa)
Validation / Design Validation	Defined as means establishing by objective evidence that device specifications (here software) conform with user needs and intended use(s). Planning, verification, traceability, configuration management, and many other aspects of good software engineering. Documentation	21 CFR 820.3(z)(2)
Process Validation	Process Validation is defined as the. collection and evaluation of data, from the. process design stage throughout. production, which establishes scientific evidence that a process is capable of consistently delivering quality products.	21 CFR 820.3(z)(1)
The Least Burdensome Approach	Defined as the minimum amount of information necessary to adequately address a relevant regulatory question or issue through the most efficient manner at the right time	Link to guidance document
Injuries / Serious Injuries	Definition of a serious injury is life threatening, or results in permanent impairment of a body function or permanent damage to a body structure; or necessitates medical or surgical intervention to preclude permanent impairment of a body function or permanent damage to a body structure.	21CFR803.3(bb)(1) and (2)
Level of Concern (Minor, Moderate, Major)	Level of Concern should be driven by the hazard analysis in the absence of mitigations, regardless of the effects of the mitigations on the individual hazards. Major if a failure or latent flaw could directly result in death or serious injury to the patient or operator. Moderate if a failure or latent design flaw could directly result in minor injury to the patient or operator. Minor if failures or latent design flaws are unlikely to cause any injury to the patient or operator.	https://www.regulations.gov/docket/FDA-2020-D-0957
Documentation	device-specific guidance; design description of the device, documentation of how the design was implemented, demonstrate design implementation testing, identified hazards and managed risks, traceability to link design, implementation, testing, and risk management	https://www.regulations.gov/docket/FDA-2020-D-0957
Software Description	comprehensive overview of the device features that are controlled by software, and describe the intended operational environment : programming language, hardware platform, operating system (if applicable), use of Off-the-Shelf software (if applicable)	https://www.regulations.gov/docket/FDA-2020-D-0957
Device Hazard Analysis / Risk Management Summary	Identification of the hazardous event, severity of the hazard, cause(s) of the hazard, method of control (e.g., alarm, hardware design), corrective measures taken (including an explanation of the aspects of the device deisng/requirments that eliminate, reduce, or warn of a hazardouse event), and verification that the method of control was implemented correctly	ISO 14971
Software Requirements Specification (SRS)	Hardware Requirements, Programming Language Requirements, Interface Requirements, Software Performance and Functional Requirements	https://www.regulations.gov/docket/FDA-2020-D-0957
Revision Level History	History of software revisions	https://www.regulations.gov/docket/FDA-2020-D-0957
Unresolved Anomalies (Bugs or Defects)	Problem, Impact on device performance, any plans or timeframes for correcting the problem (where appropriate)	https://www.regulations.gov/docket/FDA-2020-D-0957
Software Change Management	Design, development, testing, and version control of revisions to the software	https://www.regulations.gov/docket/FDA-2020-D-0957<br>https://www.regulations.gov/docket/FDA-2016-D-2021<br>https://www.regulations.gov/docket/FDA-2016-D-2021
Change Management Biological Product	Changes to an Approved Application: Biological Products	https://www.fda.gov/regulatory-information/search-fda-guidance-documents/changes-approved-application-biological-products
Change Management NDA/ANDA	Changes to an Approved NDA or ANDA	https://www.fda.gov/regulatory-information/search-fda-guidance-documents/changes-approved-nda-or-anda
Software of Unknown Pedigree (SOUP)	Software contained in a Software Device may have been obtained by the submitter from a third party	https://en.wikipedia.org/wiki/Software_of_unknown_pedigree<br>https://en.wikipedia.org/wiki/IEC_62304
Combined Products	Drug-device and biologics-device combinations	https://www.fda.gov/combination-products/about-combination-products/frequently-asked-questions-about-combination-products
Virus Protection Software	Antivirus products work by detecting, quarantining and/or deleting malicious code, to prevent malware from causing damage to your device. Modern antivirus products update themselves automatically, to provide protection against the latest viruses and other types of malware.	https://www.fda.gov/medical-devices/digital-health-center-excellence/cybersecurity
Interfaces, Networking, and Network Infrastructure	Network infrastructure is the hardware and software that enables network connectivity and communication between users, devices, apps, and the internet.	https://www.fda.gov/files/about%20fda/published/Modernization_in_Action_2022.pdf
Architecture Design Chart	An architectural diagram is a visual representation that maps out the physical implementation for components of a software system. It shows the general structure of the software system and the associations, limitations, and boundaries between each element.	https://www.regulations.gov/docket/FDA-2020-D-0957
Software Design Specification	A software design document—sometimes called software design specification—is a detailed plan for developing a piece of software. An SDD should outline the finished software's functionality (specs) and your team's plans to build it (timeline, goals, etc.).	https://www.regulations.gov/docket/FDA-2020-D-0957
Traceability Analysis	The analysis of the relationships between two or more products of the development process conducted to determine that objectives have been met or that the effort represented by the products is completed.	https://www.regulations.gov/docket/FDA-2020-D-0957
Software Development Environment Description	the development environment is a workspace with a set of processes and programming tools used to develop the source code for an application or software product.	https://www.regulations.gov/docket/FDA-2020-D-0957
Submission Content	Content of Premarket Submissions for Device Software Functions	https://www.fda.gov/regulatory-information/search-fda-guidance-documents/content-premarket-submissions-device-software-functions
Breakthrough designation	The Breakthrough Devices Program is a voluntary program for certain medical devices and device-led combination products that provide for more effective treatment or diagnosis of life-threatening or irreversibly debilitating diseases or conditions.	https://www.fda.gov/medical-devices/how-study-and-market-your-device/breakthrough-devices-program#s1
Acceptance criteria/Specification	A specification is defined as a list of tests, references to analytical procedures, and appropriate acceptance criteria which are numerical limits, ranges, or other criteria for the tests described.	https://www.fda.gov/regulatory-information/search-fda-guidance-documents/q6b-specifications-test-procedures-and-acceptance-criteria-biotechnologicalbiological-products
Conformance to specifications	Conformance to specification menas that when a device is tested according to the listed analytical procedures, will meet the acceptance criteria.	https://www.fda.gov/medical-devices/premarket-notification-510k/acceptance-checklists-510ks
Software as a Medical Device (SaMD)	Software, which on its own is a medical device – Software as a Medical Device – is one of three types of software related to medical devices.	https://www.fda.gov/medical-devices/digital-health-center-excellence/software-medical-device-samd<br>https://pubmed.ncbi.nlm.nih.gov/31818387/
Artificial Intelligence and Machine Learning (AI/ML)-Enabled Medical Devices	As technology continues to advance every aspect of health care, software incorporating artificial intelligence (AI), and specifically the subset of AI known as machine learning (ML), has become an important part of an increasing number of medical devices.	https://www.fda.gov/medical-devices/software-medical-device-samd/artificial-intelligence-and-machine-learning-aiml-enabled-medical-devices

Supplementary Table 2. Selected ISO Governance Approaches for Regulating Artifical Intelligence

**WORKING GROUPS**	
ISO/IEC JTC 1/SC 42/AG 3	AI standardization roadmapping
ISO/IEC JTC 1/SC 42/AHG 1	Dissemination and outreach
ISO/IEC JTC 1/SC 42/AHG 2	Liaison with SC 38
ISO/IEC JTC 1/SC 42/AHG 4	Liaison with SC 27
ISO/IEC JTC 1/SC 42/AHG 5	AI standardization landscape and roadmap
ISO/IEC JTC 1/SC 42/JWG 1	Joint Working Group ISO/IEC JTC1/SC 42 - ISO/IEC JTC1/SC 40: Governance implications of AI
ISO/IEC JTC 1/SC 42/JWG 2	Joint Working Group ISO/IEC JTC1/SC 42 - ISO/IEC JTC1/SC 7 : Testing of AI-based systems
ISO/IEC JTC 1/SC 42/WG 1	Foundational standards
ISO/IEC JTC 1/SC 42/WG 2	Data
ISO/IEC JTC 1/SC 42/WG 3	Trustworthiness
ISO/IEC JTC 1/SC 42/WG 4	Use cases and applications
ISO/IEC JTC 1/SC 42/WG 5	Computational approaches and computational characteristics of AI systems
**STANDARD AND/OR PROJECT**	
ISO/IEC DTS 4213.2	Information technology — Artificial Intelligence — Assessment of machine learning classification performance
ISO/IEC AWI 5259-1	Artificial intelligence — Data quality for analytics and machine learning (ML) — Part 1: Overview, terminology, and examples
ISO/IEC AWI 5259-2	Artificial intelligence — Data quality for analytics and machine learning (ML) — Part 2: Data quality measures
ISO/IEC AWI 5259-3	Artificial intelligence — Data quality for analytics and machine learning (ML) — Part 3: Data quality management requirements and guidelines
ISO/IEC AWI 5259-4	Artificial intelligence — Data quality for analytics and machine learning (ML) — Part 4: Data quality process framework
ISO/IEC AWI 5259-5	Artificial intelligence — Data quality for analytics and machine learning (ML) — Part 5: Data quality governance
ISO/IEC CD 5338	Information technology — Artificial intelligence — AI system life cycle processes
ISO/IEC AWI 5339	Information Technology — Artificial Intelligence — Guidelines for AI applications
ISO/IEC AWI 5392	Information technology — Artificial intelligence — Reference architecture of knowledge engineering
ISO/IEC AWI TR 5469	Artificial intelligence — Functional safety and AI systems
ISO/IEC AWI TS 5471	Artificial intelligence — Quality evaluation guidelines for AI systems
ISO/IEC AWI TS 6254	Information technology — Artificial intelligence — Objectives and approaches for explainability of ML models and AI systems
ISO/IEC CD 8183	Information technology — Artificial intelligence — Data life cycle framework
ISO/IEC AWI TS 8200	Information technology — Artificial intelligence — Controllability of automated artificial intelligence systems
ISO/IEC AWI TS 12791	Information technology — Artificial intelligence — Treatment of unwanted bias in classification and regression machine learning tasks
ISO/IEC AWI 12792	Information technology — Artificial intelligence — Transparency taxonomy of AI systems
ISO/IEC FDIS 22989	Information technology — Artificial intelligence — Artificial intelligence concepts and terminology
ISO/IEC FDIS 23053	Framework for Artificial Intelligence (AI) Systems Using Machine Learning (ML)
ISO/IEC DIS 23894	Information technology — Artificial intelligence — Risk management
ISO/IEC CD 24029-2	Artificial intelligence (AI) — Assessment of the robustness of neural networks — Part 2: Methodology for the use of formal methods
ISO/IEC AWI TR 24030	Information technology — Artificial intelligence (AI) — Use cases
ISO/IEC DTR 24368	Information technology — Artificial intelligence — Overview of ethical and societal concerns
ISO/IEC DIS 24668	Information technology — Artificial intelligence — Process management framework for big data analytics
ISO/IEC CD 25059	Software engineering — Systems and software Quality Requirements and Evaluation (SQuaRE) — Quality model for AI systems
ISO/IEC AWI TS 29119-11	Information technology — Artificial intelligence — Testing for AI systems — Part 11:
ISO/IEC FDIS 38507	Information technology — Governance of IT — Governance implications of the use of artificial intelligence by organizations
ISO/IEC CD 42001	Information Technology — Artificial intelligence — Management system

Supplementary Table 3. Examples of AI-tools where the Addition of Regulatory Language and/or Concepts Resulted in Documented Improvements

No.	Publication	Promise, purpose, or quote	Source	Regulatory Concept	Disconnect	Evidence for Improvement by using Regulatory Science Terms	Source
1	**IBM Watson**<br>wiki	• "*AI-assisted screening process*" for Diabetic Retinopathy (REF) (April 2020)<br>• "*Currently, there are no requirements for AI systems to be evaluated through observational clinical studies, nor is it common practice*"	ibm.com/redbook<br>ibm.com/redbook	• Intended use	• Intended use was poorly described	• Subsequent publications and strategy emphasize regulatory aspects<br>• Clarification of functionality<br>• IBM authors acknowledge critical role of regulatory aspects<br>• IBM joins ÉCLAIR guidelines, prominently feature regultory aspects	Conceptual Modeling<br>PMID: 34920529<br>PMID: 33463680<br>PMID: 33666696
2	**Google**<br>CHI paper	• "*AI-assisted screening process*" for Diabetic Retinopathy (April 2020)<br>• "*Currently, there are no requirements for AI systems to be evaluated through observational clinical studies, nor is it common practice*"	CHI paper<br>CHI paper	• Instructions for use	• Software as a Medical Device (SaMD) guidance (Dec 2018)<br>• Miscommunication between computer science and regulatory team<br>• European approach	• Appropriate instructions for use can prevent some of the published mishaps<br>• Concurrent google publication emphasize importance of regulatory aspects (May 2020)<br>• Recognition of 'context of use'	Recommendations<br>Recommendations<br>Public comment
3	**ROC curve**<br>Use and Misuse	• AI tool to predict reportability of genetic variants<br>• Performance reporting using ROC curve	Publication<br>Use and Misuse	• Indication of use (performance measures)	• Follow established statistical guidance<br>• Statistical guidance exists but is not followed	• "…Improvements to objectivity and reproducibility…"<br>• "…biases may exist in our model with regard to (…) the ethnicity or ancestry of our testing	PMID: 34979564<br>PMID: 30364844
4	**FDA 20 case studies**<br>Evidence	• Algorithmic analysis for ovarian cancer screening	Evidence<br>PMID: 12795817	• Performance measures<br>• Indication of use (Target population)<br>• Mitigation strategy	• Follow established statistical guidance<br>• Not reported (i.e., proprietary) PMID: 14996856<br>• Not included	• FDA authorized test available<br>• "Software algorithm that combines five immunoassays into a single score"<br>• One equation with 2 cut-offs by menopausal status<br>• Should not be used without an independent clinical and imaging evaluation	Decision summary<br>Decision summary<br>Decision summary<br>Decision summary

Supplementary Table 4. Established Interdisciplinary Strategies to Address Regulatory Challenges

Aspect	Approach	Reference/Source
**Review Teams**	The FDA and EMA review teams involve team members from different disciplines working collaboratively, with a common purpose, to set goals, make decisions and share resources and responsibilities.	https://www.fda.gov/industry/fda-basics-industry/guidances
		https://www.fda.gov/medical-devices/guidance-documents-medical-devices-and-radiation-emitting-products/class-ii-special-controls-documents
		https://www.ema.europa.eu/en/committees/how-committees-work
**Guidance creation**	The creation of a guidance document follows very specific paradigms that entails multiple steps that go beyond the input from immediate subject matter experts. For example, there is an intricate intra-agency review and approval process. Furthermore, the guidance document is released as a draft guidance with a public commenting period.	Link to FDA SOP
**Research by the regulators**		
	The FDA performs scientific research (e.g., Office of Science and Engineering Laboratories)	https://www.fda.gov/about-fda/cdrh-offices/office-science-and-engineering-laboratories
**Example NCTR**	**National Center for Toxicological Research; Focus Areas**	https://www.fda.gov/about-fda/science-research-nctr/nctr-research-focus-areas
	Artificial Intelligence	https://www.fda.gov/about-fda/nctr-research-focus-areas/artificial-intelligence
	Systems Biology	https://www.fda.gov/about-fda/nctr-research-offices-and-divisions/nctr-division-systems-biology
	Bio-Imaging	https://www.fda.gov/about-fda/nctr-research-focus-areas/bio-imaging
	Nanotechnology	https://www.fda.gov/about-fda/nctr-research-focus-areas/bio-imaging
	Perinatal and Maternal Research at NCTR	https://www.fda.gov/about-fda/nctr-research-focus-areas/perinatal-and-maternal-research
	Personalized Medicine	https://www.fda.gov/about-fda/nctr-research-offices-and-divisions/nctr-division-systems-biology
	NCTR biofinformatics tools	https://www.fda.gov/science-research/bioinformatics-tools
	**Regulatory Science Training**	https://www.fda.gov/about-fda/nctr-research-focus-areas/regulatory-science-training
	Facultry Research Program (NCTR)	https://www.fda.gov/about-fda/scientific-internships-fellowships-trainees-and-non-us-citizens/faculty-research-program-nctr
	Foregin National Training Program (NCTR)	https://www.fda.gov/about-fda/scientific-internships-fellowships-trainees-and-non-us-citizens/foreign-national-training-program-nctr
	Interdisciplinary Toxicology Program	https://www.fda.gov/about-fda/scientific-internships-fellowships-trainees-and-non-us-citizens/interdisciplinary-toxicology-program
	Postgraduate Research Program (NCTR)	https://www.fda.gov/about-fda/scientific-internships-fellowships-trainees-and-non-us-citizens/postgraduate-research-program-nctr
	Science Internship Program (NCTR)	https://www.fda.gov/about-fda/scientific-internships-fellowships-trainees-and-non-us-citizens/science-internship-program-nctr
	Summer Student Research Program (NCTR)	https://www.fda.gov/about-fda/scientific-internships-fellowships-trainees-and-non-us-citizens/summer-student-research-program-nctr
	Graduate Certificate in Regulatory Science	https://publichealth.uams.edu/academics/certificates/certificate-in-regulatory-science/
**Example OSEL**	The Office of Science and Engineering Laboratories (OSEL) is composed of scientists and engineers who have a broad diversity of expertise from microbiology to artificial intelligence and machine learning. We are all dedicated to promoting innovation for the development of new lifesaving medical devices.	https://www.fda.gov/about-fda/cdrh-offices/office-science-and-engineering-laboratories
	Additive Manufacturing	https://www.fda.gov/medical-devices/medical-device-regulatory-science-research-programs-conducted-osel/additive-manufacturing-program-research-additive-manufacturing-medical-devices
	Artificial Intelligence and Machine Learning (AI/ML)	https://www.fda.gov/medical-devices/medical-device-regulatory-science-research-programs-conducted-osel/artificial-intelligence-and-machine-learning-program-research-aiml-based-medical-devices
	Biocompatibility and Toxicology	https://www.fda.gov/medical-devices/medical-device-regulatory-science-research-programs-conducted-osel/biocompatibility-and-toxicology-program-research-medical-devices-biocompatibility-and-toxicology
	Cardiovascular	https://www.fda.gov/medical-devices/medical-device-regulatory-science-research-programs-conducted-osel/cardiovascular-program-research-cardiovascular-medical-devices
	Credibility of Computational Models	https://www.fda.gov/medical-devices/medical-device-regulatory-science-research-programs-conducted-osel/credibility-computational-models-program-research-computational-models-and-simulation-associated
	Digital Pathology	https://www.fda.gov/medical-devices/medical-device-regulatory-science-research-programs-conducted-osel/digital-pathology-program-research-digital-pathology-medical-devices
	Electromagnetic and Electrical Safety	https://www.fda.gov/medical-devices/medical-device-regulatory-science-research-programs-conducted-osel/electromagnetic-and-electrical-safety-program-research-electromagnetic-and-electrical-safety-medical
	Emergency Preparedness	https://www.fda.gov/medical-devices/medical-device-regulatory-science-research-programs-conducted-osel/emergency-preparedness-program-research-medical-devices-emergencies
	Human Device Interaction	https://www.fda.gov/medical-devices/medical-device-regulatory-science-research-programs-conducted-osel/human-device-interaction-program-research-human-interaction-medical-devices
	Materials Performance	https://www.fda.gov/medical-devices/medical-device-regulatory-science-research-programs-conducted-osel/materials-performance-program-research-materials-performance-medical-devices
	Medical Extended Reality	https://www.fda.gov/medical-devices/medical-device-regulatory-science-research-programs-conducted-osel/medical-extended-reality-program-research-medical-extended-reality-based-medical-devices
	Medical Imaging and Diagnostics	https://www.fda.gov/medical-devices/medical-device-regulatory-science-research-programs-conducted-osel/medical-imaging-and-diagnostics-program-research-medical-imaging-and-diagnostic-devices
	Microbiology and Infection Control	https://www.fda.gov/medical-devices/medical-device-regulatory-science-research-programs-conducted-osel/microbiology-and-infection-control-program-research-microbial-and-infection-control-medical-devices
	Microfluidics	https://www.fda.gov/medical-devices/medical-device-regulatory-science-research-programs-conducted-osel/microfluidics-program-research-microfluidics-based-medical-devices
	Neurology	https://www.fda.gov/medical-devices/medical-device-regulatory-science-research-programs-conducted-osel/neurology-program-research-neurology-medical-devices
	Ophthalmology	https://www.fda.gov/medical-devices/medical-device-regulatory-science-research-programs-conducted-osel/ophthalmology-program-research-ophthalmology-medical-devices
	Orthopedic Devices	https://www.fda.gov/medical-devices/medical-device-regulatory-science-research-programs-conducted-osel/orthopedic-devices-program-research-orthopedic-medical-devices
	Patient Monitoring and Control	https://www.fda.gov/medical-devices/medical-device-regulatory-science-research-programs-conducted-osel/patient-monitoring-and-control-program-research-patient-monitoring-and-control-devices
	Therapeutic Ultrasound	https://www.fda.gov/medical-devices/medical-device-regulatory-science-research-programs-conducted-osel/therapeutic-ultrasound-program-research-therapeutic-ultrasound-medical-devices
	**OSEL Divisions**	https://www.fda.gov/about-fda/cdrh-offices/office-science-and-engineering-laboratories
	Division of Applied Mechanics (DAM)	https://www.fda.gov/about-fda/cdrh-offices/division-applied-mechanics
	Division of Biomedical Physics (DBP)	https://www.fda.gov/about-fda/cdrh-offices/division-biomedical-physics
	Division of Biology, Chemistry, and Materials Science (DBCMS)	https://www.fda.gov/about-fda/cdrh-offices/division-biology-chemistry-and-materials-science
	Division of Imaging, Diagnostics, and Software Reliability (DIDSR)	https://www.fda.gov/about-fda/cdrh-offices/division-imaging-diagnostics-and-software-reliability
**Example ARHQ**	The Agency for Healthcare Research and Quality's (AHRQ) mission is to produce evidence to make health care safer, higher quality, more accessible, equitable, and affordable, and to work within the U.S. Department of Health and Human Services and with other partners to make sure that the evidence is understood and used. We accomplish our mission by focusing on our three core competencies.	https://www.ahrq.gov/
**Regulatory Resources**		
	Guidance database	https://www.fda.gov/regulatory-information/search-fda-guidance-documents
	Artificial Intelligence and Machine Learning (AI/ML) Software as a Medical Device Action Plan	https://www.fda.gov/medical-devices/software-medical-device-samd/artificial-intelligence-and-machine-learning-software-medical-device
	Artificial Intelligence and Machine Learning (AI/ML)-Enable Medical Devices	https://www.fda.gov/medical-devices/software-medical-device-samd/artificial-intelligence-and-machine-learning-aiml-enabled-medical-devices
	Deciding When to Submit a 510(k) for a Software Change to an Existing Device	Link to guidance
	**An FDA Artificial Intelligence (AI) Program for Toxicology at NCTR**	https://www.fda.gov/about-fda/nctr-research-focus-areas/artificial-intelligence
	AnimalGAN	https://www.fda.gov/about-fda/nctr-research-focus-areas/animalgan-initiative
	SafetAI	https://www.fda.gov/about-fda/nctr-research-focus-areas/safetai-initiative
	BERTox	https://www.fda.gov/about-fda/nctr-research-focus-areas/bertox-initiative
	PathologAI	https://www.fda.gov/about-fda/nctr-research-focus-areas/pathologai-initiative
	Medical Device Databases	https://www.fda.gov/medical-devices/device-advice-comprehensive-regulatory-assistance/medical-device-databases
	FDA product code classification database	https://www.fda.gov/medical-devices/classify-your-medical-device/product-code-classification-database
	Catalogue of Regulatory Science Tools	https://www.fda.gov/medical-devices/science-and-research-medical-devices/catalog-regulatory-science-tools-help-assess-new-medical-devices
	Multi-Reader, Multi-Case Analysis Methods (iMRMC)	https://cran.r-project.org/web/packages/iMRMC/index.html
	Software as a Medical Device (SaMD)	https://www.fda.gov/medical-devices/digital-health-center-excellence/software-medical-device-samd
	Structured Product Labeling Commercial Software and Conversion Vendors and FDA-Regulated Company Self-Generated SPL Software	https://www.fda.gov/industry/fda-data-standards-advisory-board/structured-product-labeling-resources
	Device Software Functions Including Mobile Medical Applications	https://www.fda.gov/medical-devices/digital-health-center-excellence/device-software-functions-including-mobile-medical-applications
	Medicines and Healthcare Products Regulatory Agency (MHRA), software flow chart	Link to flowchart
**Programs and Initiatves (Selection)**		
	The Medical Device Development Tool (MDDT) program	https://www.fda.gov/medical-devices/medical-device-development-tools-mddt
	Network of Expert (NoE)	https://www.fda.gov/about-fda/center-devices-and-radiological-health/network-experts-program-connecting-fda-external-expertise
	Digital Health Center of Excellence (DHCoE)	https://www.fda.gov/medical-devices/digital-health-center-excellence
	The Medical Device Innovation Consortium (MDIC)	https://mdic.org/
	Critical Path Innovation Meetings (CPIM)	https://www.fda.gov/drugs/new-drugs-fda-cders-new-molecular-entities-and-new-therapeutic-biological-products/critical-path-innovation-meetings-cpim
**Collaborative Communities**	The FDA currently participates as a member of several collaborative communities, which have been established and are managed and controlled by external stakeholders.	https://www.fda.gov/about-fda/cdrh-strategic-priorities-and-updates/collaborative-communities-addressing-health-care-challenges-together
	Toolkit	https://www.fda.gov/media/116467/download
	Collaborative Community on Ophthalmic Imaging	https://cc-oi.org/?
	National Evaluation System for health Technology Coordinating Center (NESTcc) Collaborative Community	https://nestcc.org/nestcc-one-of-the-first-collaborative-communities-with-fda-participation/
	Standardizing Laboratory Practices in Pharmacogenomics Initiative (STRIPE) Collaborative Community	http://www.stopadr.org/stripe
	International Liquid Biopsy Standardization Alliance (ILSA)	https://fnih.org/our-programs/biomarkers-consortium/programs/ilsa
	Xavier Artificial Intelligence (AI) World Consortium	https://www.xavierhealth.org/ai-summit-registration/
	Case for Quality Collaborative Community	https://mdic.org/program/case-for-quality/
	Heart Valve Collaboratory (HVC)	https://www.heartvalvecollaboratory.org/
	Wound Care Collaborative Community	https://www.woundcarecc.org/
	Pathology Innovation Collaborative Community (PICC)	https://pathologyinnovationcc.org/
	RESCUE (REducing SuiCide Rates Amongst IndividUals with DiabEtes) Collaborative Community	https://www.rescuediabetes.com
	MedTech Color Collaborative Community	https://medtechcolor.org/collaborative-community/
	Digital Health Measurement Collaborative Community (DATAcc)	https://datacc.dimesociety.org/
